# Association between plant-based diets and metabolic syndrome in obese adults from Iran: a cross-sectional study

**DOI:** 10.1186/s12902-023-01358-7

**Published:** 2023-05-16

**Authors:** Mahdi Vajdi, Arash Karimi, Ayda Zahiri Tousi, Babak Hosseini, Zeinab Nikniaz, Mahdieh Abbasalizad Farhangi

**Affiliations:** 1 Student Research Committee, Department of Community Nutrition, School of Nutrition and Food Sciences, Isfahan University of Medical Sciences, Isfahan, Iran; 2 Department of Community Nutrition, Faculty of Nutrition, Tabriz University of Medical Sciences, Tabriz, Iran; 3 Razavi Cancer Research Center, Razavi Hospital, Imam Reza International University, Mashhad, Iran; 4 Department of Surgery, School of Medicine, Laparoscopy Research Center, Shiraz University of Medical Sciences, Shiraz, Iran; 5 Liver and Gastrointestinal Diseases Research Center, Tabriz University of Medical Sciences, Tabriz, Iran; 6 Tabriz Health Services Management Research Center, Tabriz University of Medical Sciences, Attar Neyshabouri, Daneshgah Blv, Tabriz, Iran

**Keywords:** Plant-based diet, Metabolic syndrome, Obesity, Plant-based diet index

## Abstract

**Background:**

Metabolic syndrome (MetS) is a common chronic disease with several complications. Given that, studies on the association of plant-based diet indices (PDIs) with risk of MetS among adults with obesity, are limited, we aimed to examine the association between PDIs (including overall PDI, healthy PDI (hPDI), unhealthy PDI (uPDI)) and MetS in Iranian adults with obesity.

**Methods:**

In Tabriz, Iran, a total of 347 adults between the ages of 20 and 50 participated in this cross-sectional research study. We created an overall PDI, hPDI, and uPDI from validated semi-quantitative food-frequency questionnaire (FFQ) data. To investigate the association between hPDI, overall PDI, uPDI, and MetS and its components, a binary logistic regression analysis was performed.

**Results:**

The average age was 40.78 ± 9.23 years, and the average body mass index was 32.62 ± 4.80 kg/m^2^. There was no significant association between overall PDI (OR: 0.87; 95% CI: 0.54–1.47), hPDI (OR: 0.82; 95% CI: 0.48–1.40), and uPDI (OR: 0.83; 95% CI: 0.87–2.46) with MetS, even after adjustment for confounders. Moreover, our findings showed that participants with the highest adherence to uPDI had a higher chance of hyperglycemia (OR: 2.50; 95% CI: 1.13–5.52). Also, this association was significant in the first (OR: 2.51; 95% CI: 1.04–6.04) and second (OR: 2.58; 95% CI: 1.05–6.33) models, after controlling for covariates. However, in both adjusted and crude models, we did not find a significant association between hPDI and PDI scores and MetS components such as high triglyceride, high waist circumference, low High-density lipoprotein cholesterol, raised blood pressure, and hyperglycemia. Moreover, those in the top tertile of uPDI had higher fasting blood sugar and insulin levels when compared with those in the first tertile, and subjects in the last tertile of hPDI compared with participants in the first tertile had lower weight, waist-to-hip ratio, and fat-free mass.

**Conclusion:**

We found a direct significant association between uPDI and odds of hyperglycemia in the whole population of study. Future large-scale, prospective studies on PDIs and the MetS are necessary to confirm these findings.

## Introduction

**Fig. 1:**
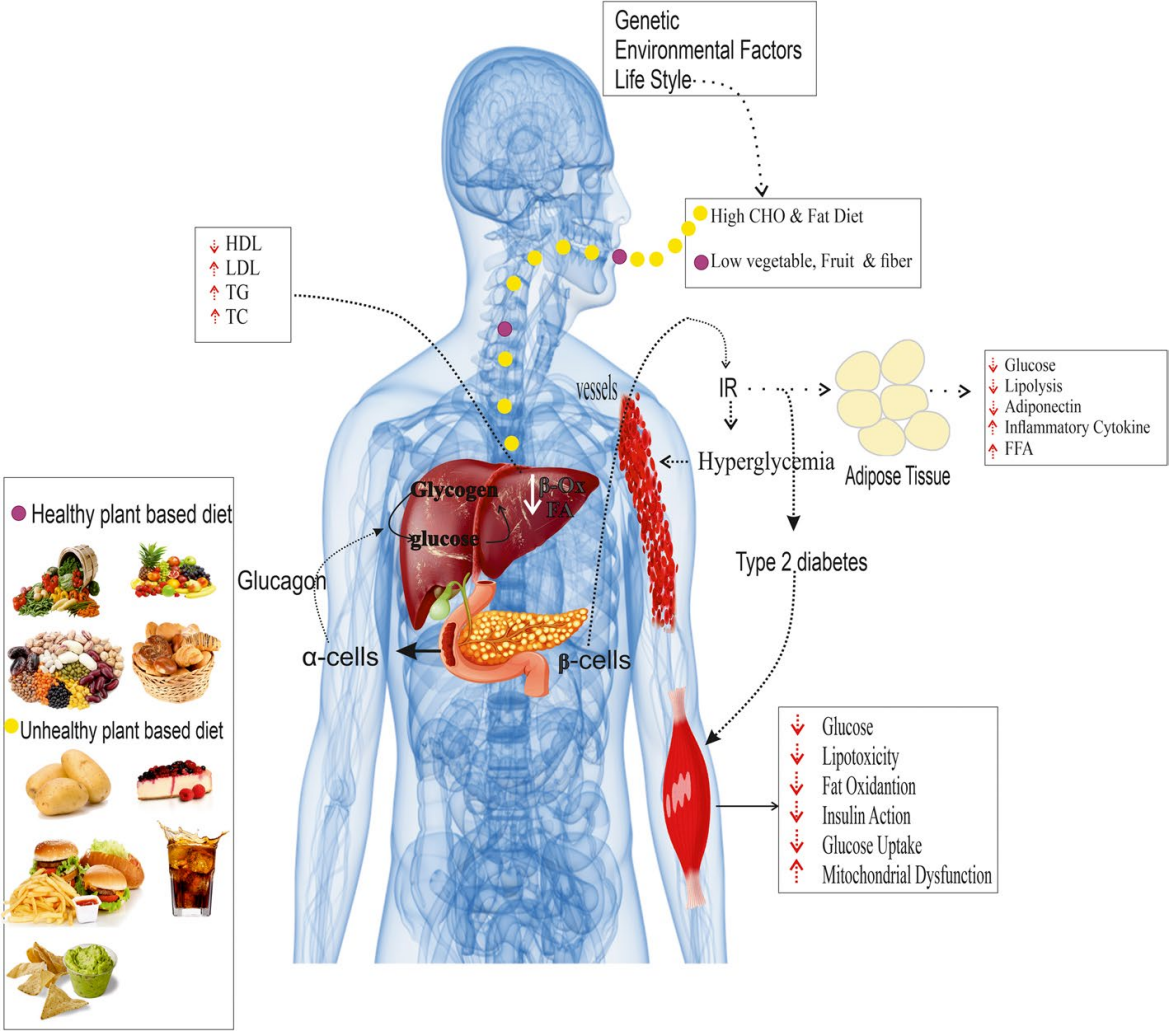
Mechanisms highlighting MetS and hyperglycemia pathophysiology

Metabolic syndrome (MetS) is a growing concern for the health and well-being of adults worldwide. MetS is linked to a number of conditions, including abdominal obesity, endothelial dysfunction, glucose intolerance, dyslipidemia, insulin resistance, and hypertension (HTN), which are directly associated with an increased risk of type 2 diabetes (T2DM) and cardiovascular disease (CVD) [1–3]. Its prevalence in the world is estimated to be around 3.3% of the whole population and 29.2% of the population obesity [4]. Furthermore, the risk of diabetes and MetS has increased five-fold in people with obesity compared with normal-weight people [5]. Current studies indicate that 33.7% of Iranians over the age of 20 have MetS, with women being more affected than men (42% versus 24%) [6]. MetS is a multifactorial condition caused by the complex interaction of lifestyle, environmental and genetic variables (Fig. 1) [7].

The establishment of healthy dietary patterns is one of several important factors that influence MetS status. Therefore, educating and encouraging adults to improve their diet quality by increasing the intake of healthy plant-based diets could be useful to prevent and treatment of MetS and its complications [8, 9]. According to certain research, patients who limit their intake of animal-based meals have positive metabolic profiles, including lower blood pressure, lower fasting blood sugar (FBS), and lower body mass index (BMI) [10, 11], but others showed no association [12, 13] or contrary relations [14–16]. The majority of these previous studies focus on the effects of a vegetarian diet, which is a subset of a plant-based diet (PBD). In addition, the consumption of plant foods, particularly less-healthy plant meals such as those high in processed carbohydrates or sugar processed carbs, has not been considered.

New plant-based indices (PDIs) have been offered as a strategy for dominating this field. A PDI that highlights intake of all plant foods while minimizing intake of animal foods; a healthy plant-based diet index (hPDI) that prioritizes consumption of plant foods associated with enhanced health outcomes, such as vegetables, fruits, and whole grains [17–19]. An unhealthful plant-based diet index (uPDI) focuses on the intake of unhealthy plant foods [19]. Numerous studies employing these indices have shown that stronger adherence to the hPDI, PDI, and the pro-vegetarian diet index is related to a lower risk of HTN, T2DM, and CVD, as well as reduced weight gain [19–21]. In an observational cohort study done by kim et al. [22] on MetS patients in South Korea. The results of this study illustrated that consumption of healthier plant foods (vegetables, fruits, nuts, whole grains, tea, coffee, and legumes) compared to relatively fewer healthy plant foods (potatoes, sugar-sweetened beverages, sweets, refined grains, salty foods) useful for prevention of MetS.

Besides, previous studies have also indicated that a plant-rich diet has been associated with MetS [23, 24], but the association of PDIs with MetS and its components in Iranian adults with obesity has been not yet studied and the link between PDIs and MetS remains unclear in obese adults. This cross-sectional study focuses mainly on the importance of PDIs as a modifiable risk factor for MetS. Therefore, in the present study, we aimed to explore the association of PDIs (including overall PDI, hPDI, uPDI) to MetS and its components in Iranian adults with obesity.

## Method

### Patients participating in the study

This cross-sectional study included 347 obese adults (58.2% men and 41.8% women). When estimating sample size, the dependence between food quality indices and obesity was considered a crucial dependent variable. The sample size was projected to be 340 using the G-power program with a correlation coefficient (r) of 0.25, α = 0.05, and power of 80% [25, 26]. Using the approach of convenience, samples were obtained via announcements. All research participants were questioned by a competent nutritionist. The participants in this study were obese adults with metabolic syndrome aged 20 to 50 years with a BMI of 30 to 40 kg/m^2^. Under the age of 18, people with a history of weight change of 5 kg or more in the last six months, pregnant women, lactating women, acute inflammatory disease, people with chronic diseases namely cancer, cardiovascular diseases, diabetes, liver or kidney diseases or chronic infections, thyroid diseases, people with a history of alcohol or drug abuse and users of slimming drugs were excluded from the study. This research was approved by the ethics committee of Tabriz University of Medical Sciences (IR.TBZMED. REC.1400.454 and grant number: 72003). Before participating in the trial, all patients provided written consent after being fully informed.

### Anthropometric assessment and general features

Nutritionists collected information about participants’ gender, age, educational level, smoking, and matrimonial status via questionnaires. House ownership, family size, educational attainment, and employment status were each collected via face-to-face interviews as independent indicators of individuals’ socioeconomic status. The gathered total score was divided into three classes: high, medium, and low based on socioeconomic status tertiles [27]. Using a validated self-administered Depression, Anxiety, and Stress Scale-21 Items questionnaire, the degree of depression was evaluated [28, 29]. Body weight was determined using a Seca (Germany) scale with a resolution of 0.1 kg, and height was determined using a stadiometer with a resolution of 0.1 cm. The waist circumference (WC) was measured using a constant tension tape in a standing position halfway between the iliac crests and lower rib borders. The BMI was calculated by dividing weight by the square of height (kg/m^2^). To get the waist-hip ratio (WHR), the WC was divided by the hip circumference. Using a BIA analyzer (Tanita, BC-428, Tokyo, Japan), fat mass (FM) and fat-free mass (FFM) were defined. Patients’ diastolic and systolic blood pressure (DBP and SBP) were measured twice after 15 min of sitting using a conventional mercury sphygmomanometer, and their average was determined.

### Description of MetS

In our study MetS was defined when three or more of the following components were met [30]: FBS ≥ 100 mg/dl or using anti-diabetic drugs; High-density lipoprotein cholesterol (HDL-C) < 50 mg/dl in women and < 40 mg/dl in men; TG ≥ 150 mg/dl or use of anti-lipid drugs; SBP ≥ 130 mmHg or DBP ≥ 85 mmHg or use of antihypertensive drugs and WC ≥ 95 cm for both genders, according to the new cut-off points for the Iranian adult population [31].

### Physical activity (PA) assessment

The International PA Questionnaire-Short Form (IPAQ-SF) was used to test PA levels in official Persian [32, 33]. This questionnaire has already been translated and validated for the Iranian adult population. Based on the information supplied by the people, the MET value was computed by taking into consideration the kind, quantity, and duration of weekly activities. At least 8.0 MET was regarded as intense physical activity, 4.0 MET as moderate, and walking at 3.3 MET [34].

### Dietary evaluation and determination of the plant-based diet index

Using a validated semi-quantitative food frequency questionnaire (FFQ) containing 147 food items, dietary intake information was collected [35]. Nutritionists with expertise in the field requested patients who participated in the research to pick the number of servings and the frequency with which they consumed each item over the previous year, monthly, weekly, yearly, or daily. A home scale was used to convert the portion sizes into grams. We utilized the Food Composition Table (FCT) from the USDA for our nutritional and energy study since the Iranian FCT does not give too much information [36]. These were converted to daily intakes and dietary information was utilized to develop three types of PDIs: an overall PDI, a high PDI, and a low PDI. We created 18 food groups based on nutrient and culinary similarities among healthy plant foods (whole grains, vegetables, fruits, tea, nuts, legumes, or coffee), fewer healthy plant foods (potatoes, sugar-sweetened beverages, refined grains, desserts, sweets, or fruit juices), and animal foods (fish or seafood, eggs, meat, dairy, other animal-based foods). The sorting of mixed-composition foods was based on the component that predominated. According to their food intake, participants were sorted into quintiles, which were then assigned negative or positive scores. Subjects scoring in the top quintile of a given food group were awarded a 5, while those scoring in the bottom quintile were awarded a 1. This scoring pattern was flipped by employing inverse scores. On the PDI, plant-based food categories received positive values, but animal-based food categories received negative values. On the hPDI, healthier plant-based food groups were assigned positive values, whereas less healthy plant-based food groups and animal-based food groups were assigned negative values. Positive values were assigned to less healthy plant food categories for the uPDI, whereas negative values were assigned to healthy plant food categories and animal food groups. The indices were computed by aggregating the scores for the 18 food categories.

### Biochemical assessment

Following a fast of 12 h, venous blood was drawn from all participants, centrifuged for 10 min at 3000 rpm, 4 °C, and frozen at -75 °C until analysis. Total cholesterol (TC), triglyceride (TG), FBS, and HDL-C levels were all tested using a commercially available kit (Pars Azmoon, Tehran, Iran). Serum low-density lipoprotein-cholesterol (LDL-C) was calculated using the Friedewald technique [37]. ELISA was used based on the manufacturer’s instructions to measure serum levels of insulin (BTL, Shanghai City, China).

### Statistical analyses

We analyzed the data using version 21 SPSS (Inc., Chicago, IL). P-values below 0.05 were used to establish the statistical significance of the study. Tertiles of PBD indices were utilized to classify every topic. As the reference class, the first tertile of PBD indices was used. Mean and standard deviation (SD) were used to describe quantitative data, whereas percentages were employed to express qualitative factors. One-way analysis of variance (ANOVA) and Chi-squared tests were used to compare quantitative and categorical variables across tertiles of PBD indices, respectively. The odds ratio (OR) and 95% confidence interval (CI) for MetS and its components across tertiles of PBD indices were determined utilizing binary logistic regression with adjusted models. In the first model, adjustments were made for education, sex, marital status, age, smoking status, occupation, and physical activity [38, 39]. Energy consumption was modified further in the second model.

## Results

### Participant characteristics

**Fig. 2:**
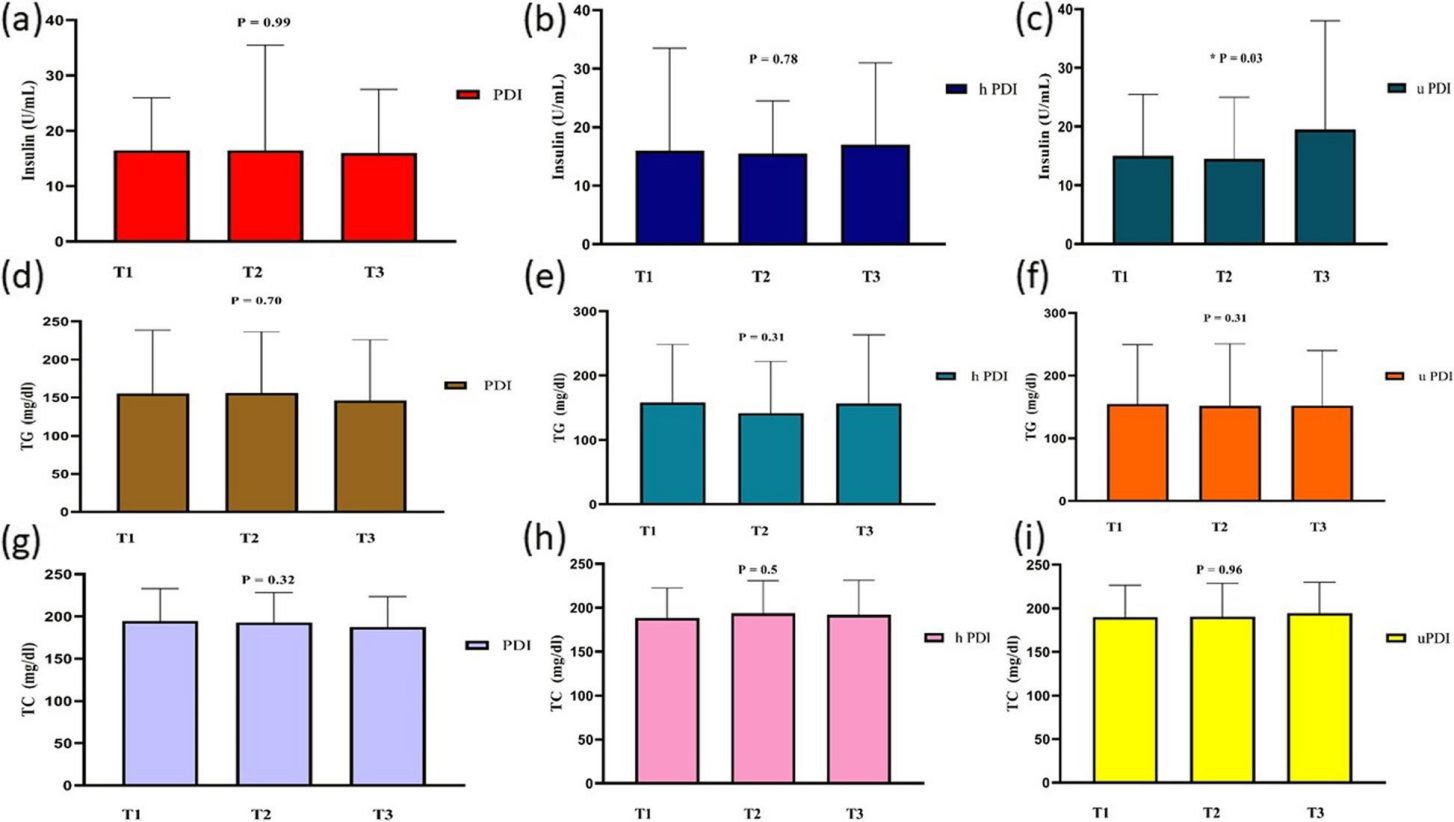
Metabolic variables (TG, TC, and insulin) of participants across tertiles of PDI, hPDI, and uPDI

**Fig. 3:**
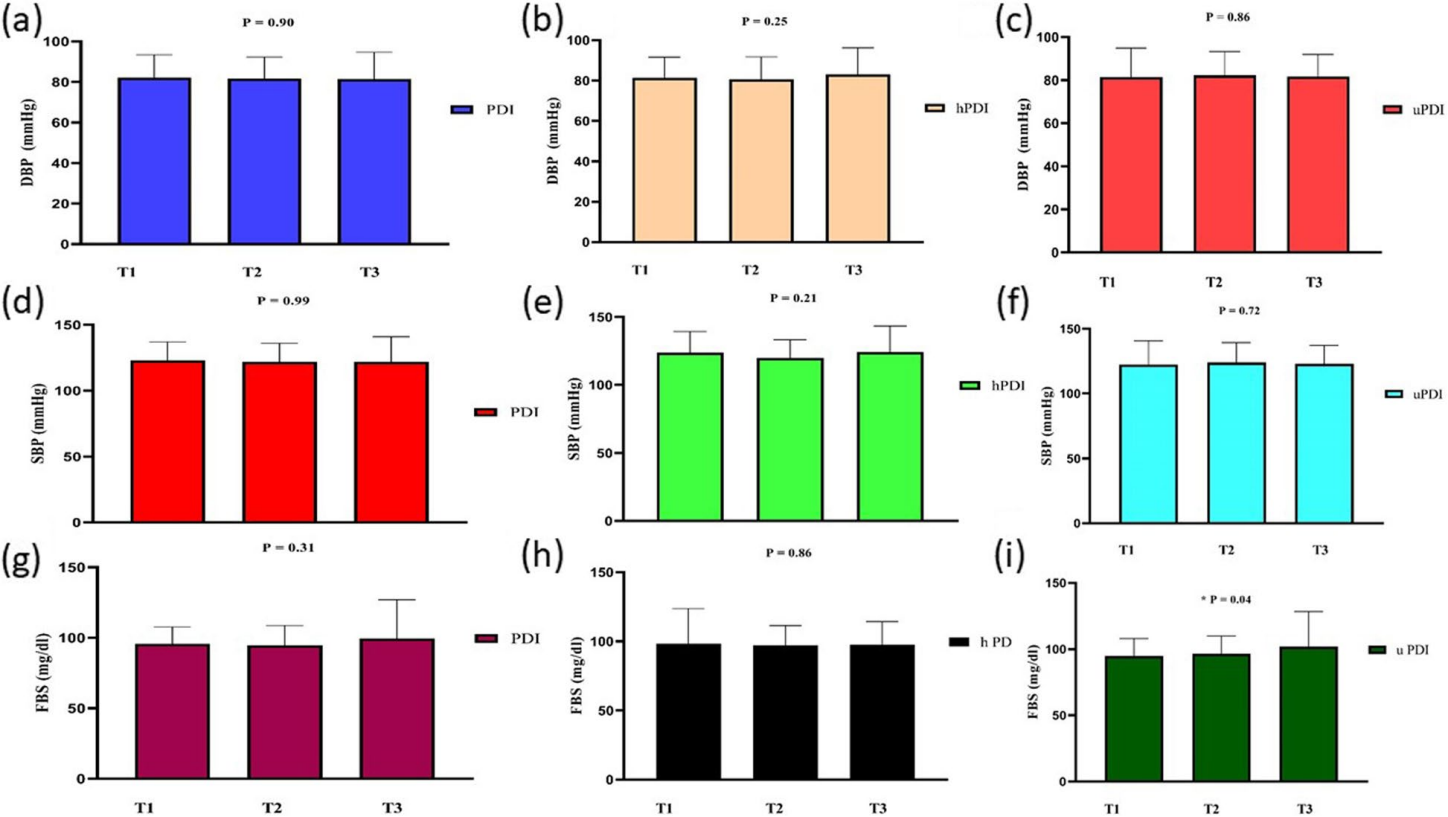
Metabolic variables (FBS, DBP, and SBP) of participants across tertiles of PDI, hPDI, and uPDI

**Fig. 4:**
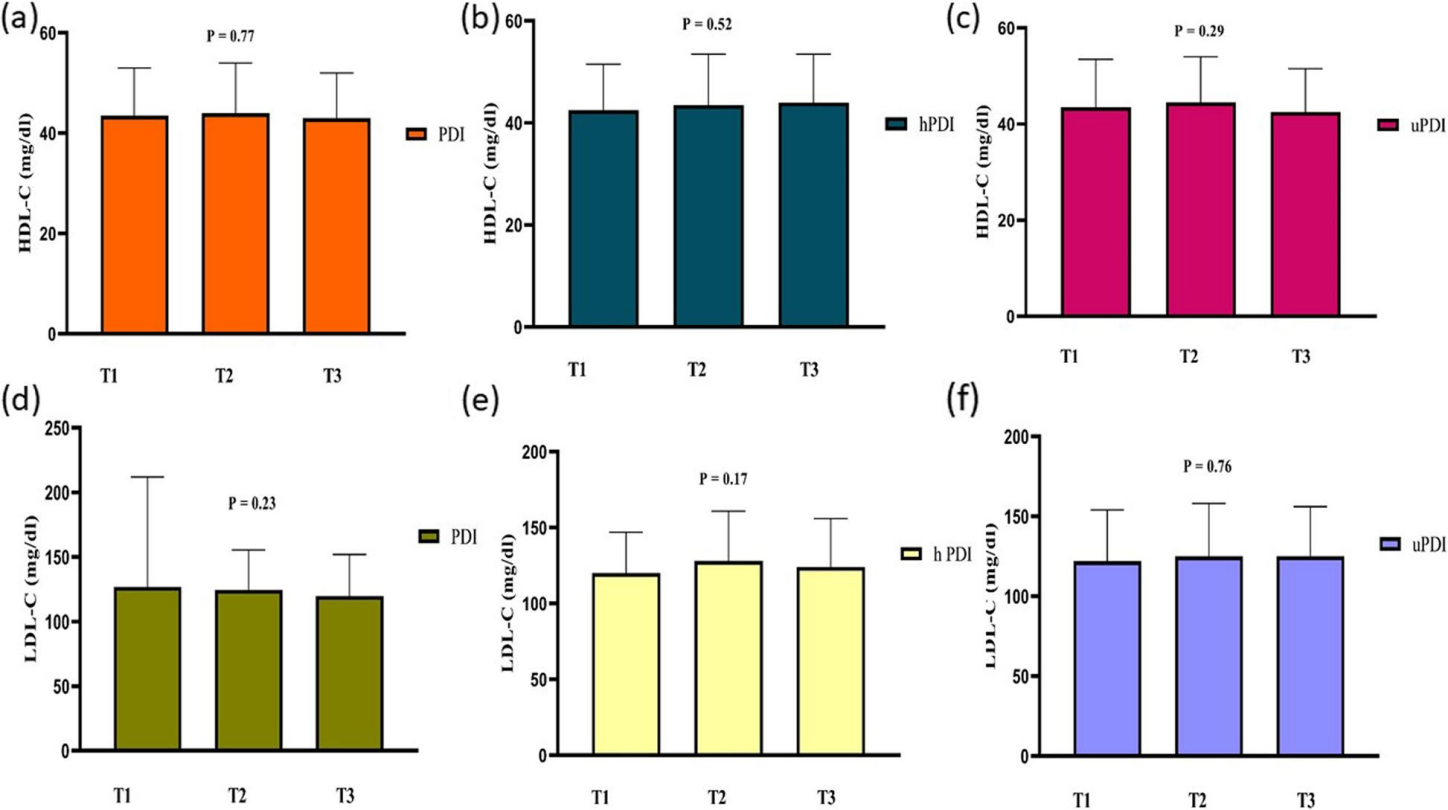
Metabolic variables (HDL-C, and LDL-C) of participants across tertiles of PDI, hPDI, and uPDI

General characteristics of the individuals across tertiles of hPDI, PDI, and uPDI are accessible in Table 1. The study included 347 participants (202 males and 145 females) with obesity, and the mean ± SD age of the subjects was 40.78 ± 9.23 years and the mean ± SD of BMI was 32.62 ± 4.80 kg/m^2^. The prevalence of MetS in our participants was 40.82%. Although most baseline variables were similar in the tertiles of PDI, participants in the last tertile of PDI compared with participants in the first tertile had higher WHR and higher intake of fat, carbohydrate, and protein (*P* < 0.05). Participants with the lowest hPDI score were significantly younger (*P* = 0.01) than those with the highest score. They had also lower weight (*P* = 0.04), WHR (*P* = 0.04), FFM (*P* = 0.03), and intake of fat, carbohydrate, and protein (*P* < 0.05). Participants in the last tertile of hPDI compared with participants in the first tertile were older and had lower weight, WHR, FFM, and a lower intake of fat, carbohydrate, and protein (*P* < 0.05). Moreover, there were significant differences in gender, socioeconomic status, and education level across tertiles of hPDI (*P* < 0.05). Participants in the last tertile of uPDI compared with participants in the first tertile had a higher intake of fat, carbohydrate, and protein (*P* < 0.05). No further differences were found in the general characteristics of participants among tertiles of uPDI (*P* > 0.05). As shown in Figs. 2, 3 and 4, we did not observe any statistically significant difference in biochemical parameters across tertiles of PDI, hPDI (*P* > 0.05). However, those in the top tertile of uPDI had higher FBS and insulin concentrations when compared with those in the bottom tertile (*P* < 0.05).

**Table 1:** The baseline characteristics of study population across tertiles of PDI, hPDI, and uPDI in a cross-sectional study of MetS in Iran (*n* = 347)

Variable	PDI			*P*	hPDI			*P*	uPDI			*P*
	**Q1** (*n* = 110)	**Q2** (*n* = 119)	**Q3** (*n* = 118)		**Q1** (*n* = 110)	**Q2** (*n* = 119)	**Q3** (*n* = 118)		**Q1** (*n* = 110)	**Q2** (*n* = 119)	**Q3** (*n* = 118)	
Age (years)	40.37 ± 8.77	41.03 ± 10.08	40.91 ± 8.81	0.84	38.17 ± 9.03	40.39 ± 8.94	43.30 ± 9.05	**0.01**	41.78 ± 9.48	40.92 ± 8.83	39.46 ± 9.28	0.16
Gender (male )	61(55.5)	65(54.6)	76(64.4)	0.24	77(72)	69(61.6)	56(43.8)	**0.01**	75(60)	64(55.7)	63(58.9)	0.78
WC (cm)	106.34 ± 9.68	105.67 ± 9.11	108.9.93	0.08	107.42 ± 8.97	106.69 ± 9.75	106.33 ± 10.06	0.68	107.63 ± 10.43	106.84 ± 9.38	105.73 ± 8.84	0.32
BMI(kg/m^2^)	32.42 ± 4.97	32.37 ± 4.53	33.07 ± 4.93	0.45	32.28 ± 4.89	32.68 ± 4.91	32.86 ± 4.66	0.64	33.06 ± 4.83	32.74 ± 4.72	31.98 ± 4.84	0.21
Weight (kg)	92.02 ± 15.27	91.13 ± 13.65	93.18 ± 14.46	0.56	94.50 ± 14.48	92.40 ± 14.44	89.86 ± 14.16	**0.04**	93.57 ± 14.73	92.34 ± 14.17	90.15 ± 14.28	0.19
WHR (m)	0.93 ± 0.08	0.91 ± 0.07	0.94 ± 0.07	**0.01**	0.95 ± 0.07	0.93 ± 0.06	0.92 ± 0.07	**0.04**	0.93 ± 0.06	0.93 ± 0.08	0.93 ± 4.84	0.82
FM (kg)	34.09 ± 9.26	33.13 ± 8.78	34.159.41	0.78	32.22 ± 10.11	33.37 ± 8.45	35.59 ± 8.74	0.11	35.51 ± 8.83	34.56 ± 9.95	33.37 ± 8.69	0.73
FFM (kg)	61.48 ± 13.18	61.06 ± 11.72	63.88 ± 12.18	0.36	65.28 ± 11.70	62.51 ± 12.67	59.42 ± 12.10	**0.03**	62.69 ± 12.81	62.63 ± 11.76	61.29 ± 12.54	0.78
**Physical activity (%)**				0.42				0.19				0.11
Low	50(45.6)	51(43.3)	63(53.5)		41(37.5)	56(48.5)	66(56.1)		44(40.3)	73(61.7)	51(42.9)	
Moderate	37(33.3)	38(31.7)	23(19.7)		37(33.9)	27(22.7)	32(27.3)		34(30.6)	28(23.3)	34(28.6)	
High	23(21.1)	30(25)	32(26.8)		31(28.6)	34(28.8)	20(16.7)		32(29.2)	18(15)	34(28.6)	
**Marital status (%)**												
Married	96(87.3)	96(80.7)	104(88.1)	0.51	92(86)	94(83.9)	110(85.9)	0.98	101(80.8)	101(87.8)	94(87.9)	0.39
**SES (%)**				0.45				**0.02**				0.51
Low	4(3.5)	0(0)	5(4.3)		0(0)	0(0)	10(7.7)		2(1.4)	3(3.3)	4(3.6)	
Middle	54(49.1)	71(60)	59(50)		53(48.2)	56(47)	74(63.1)		53(47.9)	62(51.7)	72(60.7)	
High	52(47.4)	24(40)	54(45.7)		57(51.8)	63(53)	34(29.2)		56(50.7)	54(45)	42(35.7)	
**Depression (%)**				0.61				0.68				0.68
Normal	77(70.2)	89(75)	92(77.5)		89(80.4)	88(74.2)	82(69.7)		81(74)	85(71.7)	91(76.8)	
Mild	25(22.8)	18(15)	9(12.7)		15(14.3)	20(16.7)	21(18.2)		15(13.9)	26(21.7)	17(14.3)	
Moderate	8(7)	6(10)	12(9.9)		6(5.4)	11(9.1)	14(12.1)		12(11.1)	8(6.7)	10(8.9)	
**Education (%)**				0.71				**0.01**				0.71
Illiterate	11(10.6)	4(3.3)	10(8.5)		0(0)	0(0)	4(3.1)		1(1.4)	0(0)	2(1.8)	
≤ High school/diploma	15(14)	20(16.7)	20(17.1)		16(14.3)	29(24.2)	33(27.6)		25(22.6)	24(20)	29(25)	
≥ College degree	83(75.4)	95(80)	88(74.3)		94(85.7)	90(75.8)	82(69.2)		84(76.1)	95(80)	86(73.2)	
Protein	85.19 ± 30.39	99.28 ± 32.09	114.06 ± 42.23	**0.01**	110.75 ± 38.58	98.01 ± 39.74	91.46 ± 30.65	**0.01**	78.90 ± 25.87	97.13 ± 28.34	120.61 ± 41.74	**0.01**
Carbohydrate	351.53 ± 134.11	456.92 ± 145.46	543.65 ± 171.71	**0.01**	484.53 ± 166.82	424.29 ± 162.14	448.30 ± 175.72	**0.03**	391.03 ± 143.39	433.68 ± 144.60	523.69 ± 188.34	**0.01**
Fat	83.86 ± 41.53	100.51 ± 43.38	116.35 ± 50.59	**0.01**	111.63 ± 46.78	101.97 ± 50.45	89.24 ± 41.79	**0.01**	78.14 ± 35.61	99.09 ± 46.07	121.58 ± 47.88	**0.01**

*P*-value obtained using one-way ANOVA for continuous variables and Chi-square test for categorical variables. Categorical and continuous variables data are presented as number (percent) and mean (SD)

*Abbreviations*: *BMI* body mass index, *FM* Fat mass, *FFM* Fat free mass, *SES *Socio-economic status, *PDI *plant-based diet index, *Hpdi *healthful plant-based diet index, *uPDI *unhealthful plant-based diet index

### Association between PDIs and MetS and its components

Multivariable-adjusted OR and 95% CIs for MetS and its components according to tertiles of uPDI, PDI, and hPDI are shown in Table 2. In the crude model, a higher score of uPDI was directly associated with the risk of hyperglycemia (OR: 2.50; 95% CI: 1.13–5.52). Also, this association was significant in the first (OR: 2.51; 95% CI: 1.04–6.04) and second (OR: 2.58; 95% CI: 1.05–6.33) models, after controlling for covariates. However, no significant association was found between uPDI and risk of MetS and high WC, high TC, high TG, low HDL-C, and elevated blood pressure in crude and adjusted models. No significant association was found between PDI and hPDI and risk of MetS (OR: 0.87; 95% CI: 0.54–1.47, OR: 1.46; 95% CI: 0.87–2.46, respectively) and its components. After adjustment for covariates, including age, sex, occupation, marital status, education, smoking status, physical activity, and energy intake, the association remained unchanged.

**Table 2:** Odd’s ratio (OR) and confidence interval (CI) for MetS and its components according to tertiles of PDI, hPDI, and uPDI in a cross-sectional study of MetS in Iran (*n* = 347)

Variable	PDI			*P*	hPDI				*P*	uPDI			*P*
	**Q1** (*n* = 110)	**Q2** (*n* = 119)	**Q3** (*n* = 118)		**Q1** (*n* = 110)		**Q2** (*n* = 119)	**Q3** (*n* = 118)		**Q1** (*n* = 110)	**Q2** (*n* = 119)	**Q3** (*n* = 118)	
**MetS**^**A**^													
Crude	1 (Ref.)	**0.77 (0.45–1.32)**	**0.87 (0.54–1.47)**	0.62		1 (Ref.)	**0.87 (0.50–1.51)**	**0.82 (0.48–1.40)**	0.12	1 (Ref.)	**0.87 (0.52–1.46)**	**0.83 (0.87–2.46)**	0.48
Model 1^B^	1 (Ref.)	**0.98 (0.39–2.46)**	**1.30 (0.55–3.04)**	0.52		1 (Ref.)	**0.76 (0.31–1.87)**	**0.87 (0.34–2.22)**	0.80	1 (Ref.)	**0.76 (0.22–1.77)**	**0.84 (0.34–2.07)**	0.68
Model 2^C^	1 (Ref.)	**0.89 (0.34–2.35)**	**1.12 (0.42–2.96)**	0.78		1 (Ref.)	**0.83 (0.32–2.11)**	**0.96 (0.36–2.54)**	0.96	1 (Ref.)	**0.80 (0.34–1.91)**	**0.97 (0.36–2.56)**	0.91
**High WC**													
Crude	1 (Ref.)	**0.61 (0.13–2.68)**	**0.92 (0.20–4.33)**	0.84		1 (Ref.)	**3.13 (0.58–16.84)**	**1.19 (0.32–4.36)**	0.81	1 (Ref.)	**0.52 (0.14–1.97)**	**1.58 (0.28-9.00)**	0.72
Model 1^B^	1 (Ref.)	**0.77 (0.14–4.28)**	**1.10 (0.19–6.33)**	0.86		1 (Ref.)	**3.03 (0.45–20.03)**	**0.71 (0.14–3.61)**	0.71	1 (Ref.)	**0.33 (0.07–1.58)**	**1.79 (0.25–12.48)**	0.73
Model 2^C^	1 (Ref.)	**0.74 (0.12–4.44)**	**1.03 (0.15–7.13)**	0.91		1 (Ref.)	**3.03 (0.45–20.07)**	**0.72 (0.14–3.69)**	0.72	1 (Ref.)	**0.34 (0.07–1.64)**	**1.96 (0.27–14.24)**	0.68
**High Cholesterol**													
Crude	1 (Ref.)	**0.89 (0.42–1.91)**	**0.70 (0.33–1.47)**	0.96		1 (Ref.)	**1.17 (0.68–2.38)**	**0.54 (0.24–1.20)**	0.17	1 (Ref.)	**0.60 (0.27–1.36)**	**0.47 (0.19–1.14)**	0.08
Model 1^B^	1 (Ref.)	**1.12 (0.49–2.59)**	**0.69 (0.30–1.57)**	0.37		1 (Ref.)	**1.09 (0.50–2.37)**	**0.58 (0.54–1.38)**	0.25	1 (Ref.)	**0.50 (0.21–1.22)**	**0.42 (0.16–1.09)**	0.06
Model 2^C^	1 (Ref.)	**1.03 (0.42–2.48)**	**0.60 (0.23–1.54)**	0.26		1 (Ref.)	**1.07 (0.49–2.34)**	**0.54 (0.21–1.39)**	0.24	1 (Ref.)	**0.52 (0.21–1.26)**	**0.44 (0.16–1.22)**	0.09
**High TG**													
Crude	1 (Ref.)	**1.03 (0.40–2.66)**	**1.80 (0.76–4.25)**	0.15		1 (Ref.)	**0.75 (0.31–1.76)**	**0.80 (0.34–1.88)**	0.63	1 (Ref.)	**0.94 (0.43–2.04)**	**0.26 (0.09–1.74)**	0.11
Model 1^B^	1 (Ref.)	**1.44 (0.48–4.32)**	**2.51 (0.94–6.71)**	0.05		1 (Ref.)	**0.83 (0.31–2.26)**	**0.87 (0.30–2.49)**	0.80	1 (Ref.)	**0.88 (0.35–2.19)**	**0.25 (0.07–1.79)**	0.12
Model 2^C^	1 (Ref.)	**1.32 (0.41–4.18)**	**2.20 (0.72–6.70)**	0.14		1 (Ref.)	**0.97 (0.34–2.71)**	**1.02 (0.34–3.07)**	0.95	1 (Ref.)	**0.91 (0.36–2.31)**	**0.27 (0.07–1.93)**	0.15
**Low HDL-C**													
Crude	1 (Ref.)	**0.69 (0.33–1.44)**	**0.66 (0.32–1.35)**	0.27		1 (Ref.)	**0.84 (0.41–1.71)**	**1.25 (0.61–2.56)**	0.51	1 (Ref.)	**1.29 (0.65–2.56)**	**1.58 (0.77–3.22)**	0.19
Model 1^B^	1 (Ref.)	**0.78 (0.35–1.72)**	**0.66 (0.31–1.42)**	0.29		1 (Ref.)	**0.55 (0.55–1.24)**	**0.73 (0.31–1.68)**	0.49	1 (Ref.)	**1.20 (0.57–2.53)**	**1.65 (0.76–3.58)**	0.20
Model 2^C^	1 (Ref.)	**0.82 (0.35–1.88)**	**0.72 (0.30–1.72)**	0.46		1 (Ref.)	**0.50 (0.22–1.14)**	**0.65 (0.27–1.53)**	0.38	1 (Ref.)	**1.17 (0.55–2.49)**	**1.55 (0.67–3.57)**	0.30
**Hyperglycemia**													
Crude	1 (Ref.)	**0.86 (0.37-2.00)**	**0.87 (0.38–1.99)**	0.72		1 (Ref.)	**1.10 (0.46–2.60)**	**1.07 (0.45–2.47)**	0.87	1 (Ref.)	**1.65 (0.74–3.69)**	**2.50 (1.13–5.52)**	0.28
Model 1^B^	1 (Ref.)	**1.18 (0.45–3.11)**	**1.24 (0.50–3.03)**	0.63		1 (Ref.)	**1.13 (0.44–2.92)**	**1.00 (0.36–2.72)**	0.98	1 (Ref.)	**1.16 (0.45-3.00)**	**2.51 (1.04–6.04)**	0.88
Model 2^C^	1 (Ref.)	**1.05 (0.38–2.89)**	**1.02 (0.36–2.84)**	0.97		1 (Ref.)	**1.27 (0.48–3.39)**	**1.13 (0.40–3.18)**	0.84	1 (Ref.)	**1.21 (0.46–3.19)**	**2.58 (1.05–6.33)**	0.84
**Elevated blood pressure**													
Crude	1 (Ref.)	**0.66 (0.25–1.76)**	**1.57 (0.69–3.56)**	0.21		1 (Ref.)	**1.53 (0.61–3.84)**	**1.95 (0.80–4.79)**	0.14	1 (Ref.)	**0.89 (0.39–2.03)**	**0.88 (0.38–2.04)**	0.76
Model 1^B^	1 (Ref.)	**0.80 (0.27–2.35)**	**1.76 (0.69–4.51)**	0.19		1 (Ref.)	**1.36 (0.49–3.74)**	**1.21 (0.41–3.53)**	0.75	1 (Ref.)	**0.95 (0.37–2.39)**	**1.14 (0.42–3.05)**	0.81
Model 2^C^	1 (Ref.)	**0.72 (0.23–2.20)**	**1.45 (0.49–4.27)**	0.40		1 (Ref.)	**1.61 (0.56–4.63)**	**1.46 (0.48–4.46)**	0.54	1 (Ref.)	**1.05 (0.41–2.68)**	**1.51 (0.51–4.40)**	0.47

*P* values are reported based on the multivariate multinomial logistic regression test and are considered significant at <0.05. The multivariate multinomial logistic regression was used for estimation of ORs and confidence interval (CI). ^A^ Defined as the presence of at least three of the following components: TG ≥ 150 mg/dl; WC ≥ 88 cm in women and ≥ 102 cm in men; DBP ≥ 85 mmHg or SBP ≥ 130 mmHg; HDL-C < 50 mg/dl in women and < 40 mg/dl in men and fasting glucose ≥ 100 mg/dl. ^B^ Model 1: Adjusted for age, sex, occupation, marital status, education, smoking status, and physical activity. ^C^ Model 2: Model 1 + energy intake

## Discussion

To the best of our knowledge, the current cross-sectional study is the first to examine the association between PDIs and MetS and its components in Iranian adults with obesity. In this adult sample, there was no significant association between hPDI and PDI scores and MetS and its components, whereas a high uPDI score was associated with an increased risk of hyperglycemia. This association remained significant even after considering potential covariates. Moreover, those in the top tertile of uPDI had higher FBS and insulin levels when compared with those in the first tertile and participants in the last tertile of hPDI compared with participants in the first tertile had lower weight, WHR, and FFM.

Our results were compared with those from several studies assessing the relationship between MetS risk and: (1) overall diet, which includes animal-based and plant components, or (2) vegetarian diets. In line with our study, Huo et al. [40] in a cohort study in Chinese adults also revealed no significant relationship between uPDI and MetS, however, they found an inverse association between the hPDI and MetS. They reported that participants in the highest quintile of hPDI score had a 20% lower risk of developing high WC and had a 28% lower risk of developing MetS. Compared with the findings of a study on a PBD and MetS in South Korea (KoGES cohort), when adjusting for lifestyle and demographic variables, participants with the highest score of uPDI (diets high in sugars, salted vegetables, and refined carbohydrates, and low in healthy animal and plant foods) had a 44% greater risk of having incident MetS compared to those with the lowest score of uPDI, nevertheless they did not find a relationship between MetS and hPDI [22]. In the SUN cohort study, they showed a robust negative relationship between the healthy pro-vegetarian food pattern and overweight/obesity incidence, while these were not significant for the unhealthy pro-vegetarian food pattern [41]. The vegetarian food pattern was calculated likewise to the PDIs in our study. Contrary to the results of earlier studies, this study did not support the relationship between PDIs and with risk of having MetS [42–44]. The observed results are different from findings obtained from the Persian Kavar cohort study (PKCS) conducted among healthy Iranian individuals, showing a significant inverse relationship between hPDI and MetS, low serum HDL-C, and hyperglycemia [44]. However, in line with our results, several studies conducted in South Korea have currently shown no significant association between hPDI and MetS [22, 45].

The differences between our findings and the previous research could be due to Iran’s nutrition transition as a middle-income country. The conventional Iranian cuisine comprises a variety of food items, including fruits, vegetables, dairy products, poultry, meat, fresh leafy greens, and nuts [46]. Transitioning to a Western dietary pattern may have unfavorably affected Iranians’ dietary patterns. Western diets tend to contain larger portion sizes, which can lead to excessive energy consumption and an increased risk of obesity and its related diseases [47]. Furthermore, Western diets characterized by a high intake of processed and red meat are related to a higher incidence of MetS [48, 49]. We did not find an association between hPDI and MetS or its components. However, subjects in the last tertile of hPDI compared with participants in the first tertile had lower weight, WHR, and FFM. These results were as expected, as earlier research has reported favorable relations between weight and adherence to a hPDI [20, 50]. Probably, higher plant food consumption in subjects that is already consuming a PBD may not cause clinically significant metabolic responses. Moreover, the detected associations with hPDI may have been inconsequential owing to a distinct categorization of food items in the present investigation, as certain plant-based foods that are less healthy could not be distinguished from those that are healthy. Different study designs, participants’ characteristics, geographical regions, lack of adjusting for possible confounding factors, different sample sizes, and types of bias might explain the differences between our results and those of previous research. Generally, more research using the PDI, uPDI, and hPDI might help resolve these discrepancies.

MetS, characterized by hypertension, insulin resistance, abdominal obesity, low-grade inflammation, and dyslipidemia. The biological rationale for the possible relationship of PBD on MetS risk might be as follow: lower intake of meat, animal fats, and animal-based foods and higher consumption of healthy plant- foods such as fruits, vegetables, vegetable oils, whole grains, nuts, and legumes is accompanied with higher consumption of antioxidants, polyunsaturated fatty acids, fiber, isoflavones, phytochemicals, calcium, vitamins, all of which being inversely associated with obesity and its related diseases [51]. The synergic effects of these healthy foods can have beneficial effects on MetS and its components.

One of the biological treatments that are involved in decreasing MetS and its complications is a low level of inflammation status. Low consumption of fruits, vegetables, and whole grains is related to increased inflammatory markers [52]. Findings from a meta-analysis of PBDs and its association with inflammatory markers revealed that intake of a vegetarian-based diet was related to lower CRP, and fibrinogen levels compared with those following a mixed omnivorous diet [53]. Moreover, a cross-sectional study among Iranian women with obesity also shown that higher hPBD adherence was related to lower transforming growth factor and CRP levels [54]. Fiber consumption positively changes the microbiota and increases gut barrier function and anti-inflammatory molecules [55]. Dietary fiber has been revealed to decline plasma TC by binding to bile acids and dietary cholesterol in the intestinal tract, resulting in reduced cholesterol absorption [56]. Moreover, n-3 polyunsaturated fatty acids contained in fish have been revealed to possess cardioprotective, anti-inflammatory, and hypotriglyceridemic propertie [57]. Monounsaturated fatty acids from nuts and olive oil can be anti-inflammatory in PBD, mainly by replacing saturated fat consumption [58]. Polyphenols may prevent the development of MetS by lowering plasma glucose, blood pressure, body weight, and improving dyslipidemia [59, 60]. Isoflavones, have anti-inflammatory effects and potentially protect against MetS by preventing the onset of dyslipidemia, hyperglycemia, and hypertension [61]. Furthermore, phytochemicals modify inflammation by several mechanisms, such as modulating mitogen-activated protein kinase, inhibiting arachidonic acids pathways and nuclear factor kappa-B [62]. Thomas et al. [60] reviewed the effects of different types of PBDs on weight, insulin resistance, dyslipidemia, hypertension, and low-grade inflammation. They concluded that vegan diets are effective in reducing weight and inflammatory markers, moreover, lacto-ovo- and lacto-vegetarian diets, that contain antioxidants and nutrients that improve inflammation and dyslipidemia, seem to provide positive effects for MetS.

Consumptions of dietary antioxidants might improve glucose metabolism by reducing glucose absorption and improving insulin sensitivity [63]. Furthermore, micronutrients such as vitamin C, magnesium, and potassium have been shown to improve insulin sensitivity, and blood pressure, and protect against inflammatory and oxidative stress [64–66]. Nevertheless, increased consumption of added sugars can lead to adverse effects on weight, blood pressure, lipid metabolism, and glycemic control, particularly high dietary fructose can increase hepatic de novo lipogenesis [67]. PBDs have a low energy density and low-energy food consumption is important for weight control and can decrease the incidence of abdominal obesity [68, 69]. Such a diet could have so several positive effects on health such as helping weight loss or maintenance, insulin regulation, improving lipid profile, and reducing blood pressure [17–19].

In recent years, the development of big retail supply of food has preferred energy-dense and processed foods over than fresh ones, with high salt, fat, and sugar content [70]. An increasing body of evidence shows that local food environments might affect health status [71–73]. It is consequently possible to speculate that length of a food supply chain might affect MetS and its components. Soummer et al. [70] in an observational study in Southern Italy demonstrated thate length of food supply chain plays a key role in determining the risk of MetS in a population adhering to the Mediterranean diet. Moreover, they reported that short supply chains is associated with a lower prevalence of MetS and insulin resistance is significantly more common among long supply chain subjects compared with short supply chains individuals.

### Key elements of PBDs associated with reduced risk of MetS

There may be numerous dietary components of PBDs that are working together to have a positive and significant effect on MetS. These include both food groups and nutrients that are usually lower in PBDs (meat, saturated fat, and energy intake) or higher in PBDs (fiber, vegetables, and fruits) [74]. This may be one reason why PBDs may be protective against the development of MetS. One dietary benefit of following PBDs may be consuming a diet lower in energy content, as compared with omnivorous diets. In numerous studies comparing vegetarian diets with omnivorous dietary patterns, significant weight loss has occurred in the absence of significantly different alterations in reported energy consumption between groups [75, 76]. Moreover, in a cohort study BMI was found to be highest among omnivores and lowest among vegans; however energy consumption did not significantly differ among the groups [77]. Several studies have detected that diets high in saturated fat are related to increased risk of developing MetS. Both experimental [78, 79] and observational [77, 80] studies have consistently revealed lower saturated fat consumption among people following a vegan diet, as compared with omnivores. People following vegetarian diets tend to have higher vegetable and fruit consumption than those following omnivorous diets [81, 82]. High intake of vegetable and fruit can provide individuals with important antioxidants, which may help to prevent inflammation and oxidative stress. Moreover, vegetables and fruits are a good source of fiber, which can also help with preventing MetS and CVDs [83]. A study of Spanish adults revealed that dietary fiber consumption was related to a reduction in weight and WC [84], and dietary fiber can significantly reduce energy intake. Furthermore, energy consumption may be affected by dietary fiber in particular by changes in satiety and hunger signals. Absorption of water by soluble fibers could lead to a higher amount of viscous gel formation, higher satiety, and slower gut transit time [85]. Moreover, fiber can stimulate the fecal defecation of bile acids, thus lowering serum TC and improving the lipid profile [86]. However, higher glycemic index and a load of such an unhealthy diet might cause a decrease in satiety and an increase in hunger signals [87]. Processed and red meat consumption has been related to increased risk of MetS and T2DM [88, 89]. Exclusion of meat from the diet, which occurs when an individual follows a vegetarian or vegan diet, is one potential approach to mitigate the risk of developing MetS. Even people following PBDs consume less animal protein than do omnivores, while intakes are even lower among those following PBDs [77].

## Strengths and limitations

Several strengths of this study include an adjustment for several confounding factors in the analysis, being the first study among adults with obesity, and using a validated FFQ and energy-adjusted values of all food groups for constructing PDIs. Certain plant-based foods, such as refined grains, potatoes, and sugar-sweetened beverages, have an adverse effect on health status [90, 91]. Thus, the study examined the impact of a comprehensive plant-based diet on MetS and analyzed the influence of healthy and unhealthy plant-based foods separately. However, some potential limitations also need to be kept in mind when interpreting results. The present study was carried out in a cross-sectional design, which is prone to misclassification, selection bias, recall bias, and response bias and would not allow us to infer causality. Although we used a validated FFQ, reporting of dietary consumption can still be subject to measurement error. Therefore, the results of this study may not represent all adults with obesity. Moreover, although we adjusted for several covariates, we could not control for some unknown and residual covariates, which could lead to confounding bias. Lastly, different cooking methods can have an influence on the nutritional value of plant food products, however the effects of cooking methods were not considered in the study.

## Conclusion

In conclusion, our study of adults with obesity revealed a positive correlation between elevated uPDI scores and an increased risk of hyperglycemia. The study findings indicate that there was no significant association between PDI or hPDI and MetS or its components in the adult population with obesity. Additional prospective research on the relationship between PDI and MetS is required to validate the observed associations.

The findings of the present investigation indicate a positive correlation between increased adherence to uPDI score and a higher risk of hyperglycemia. Nonetheless, comprehending this correlation could be facilitated by considering the nutritional composition. The dietary makeup of an individual with upper percentile dietary intake (uPDI) may consist of elevated consumption of plant-based foods that are deemed less healthy, including but not limited to confectioneries, fruit juices, processed grains, sugary desserts, and starches that have been sweetened with sugar. Conversely, the consumption of antioxidants and micronutrients may be comparatively lower, which could potentially have a negative impact on the development of metabolic syndrome (MetS) and its associated components. These foods have a high glycemic index and several studies have reported that high glycemic index foods increase fat storage and therefore increase visceral adiposity and weight [92]. A study in the US reported that women with higher dietary consumption of lipids and lower dietary consumption of micronutrients (vitamins A, E, C, and folate) had a raised risk of MetS [93].

## Data Availability

The datasets generated and/or analyzed during the current study are not publicly available due to some restrictions that applied by the ethical committee but are available from the corresponding author on reasonable request.
